# 慢性移植物抗宿主病诊断与治疗中国专家共识（2024年版）

**DOI:** 10.3760/cma.j.cn121090-20240611-00217

**Published:** 2024-08

**Authors:** 中华医学会血液学分会干细胞应用学组

## Abstract

慢性移植物抗宿主病（cGVHD）是异基因造血干细胞移植后常见且严重的并发症，严重影响患者的生存率和生活质量。近年来，随着新型靶向药物等新疗法的出现及临床研究的不断推进，cGVHD在诊断、预防、治疗等方面取得了显著进步。基于近年来cGVHD研究新进展及逐渐增多的循证医学证据，本共识在《慢性移植物抗宿主病（cGVHD）诊断与治疗中国专家共识（2021年版）》基础上进行了修订及更新，以更好地指导临床实践。

## Abstract (en)

Chronic graft-versus-host disease（cGVHD）is a common and severe complication following allogeneic hematopoietic stem cell transplantation, which significantly impacts patients' survival and quality of life. In recent years, notable progress has been made in the diagnosis, prevention, and treatment of cGVHD, driven by the emergence of novel therapies such as targeted drugs and the advancement of clinical research. This consensus, based on the latest developments in cGVHD research and growing data from evidence-based medicine, has been revised and updated from the “Chinese consensus on the diagnosis and management of chronic graft-versus-host disease（2021）” to better guide clinical practice.

慢性移植物抗宿主病（chronic graft-versus-host disease, cGVHD）指异基因造血干细胞移植（allo-HSCT）后，受者在重建供者免疫的过程中，来源于供者的淋巴细胞攻击受者脏器产生的临床病理综合征（包括经典型cGVHD和重叠综合征），是移植后主要并发症之一，发生率为30％～70％[1]–[2]。cGVHD发生机制复杂，临床表现多样，个体差异大，病程迁延持久。随着移植技术体系的不断完善，患者对移植后生活质量的诉求越来越高，重视cGVHD的防治非常重要，否则将影响患者的生活质量和远期生存。本共识是在《慢性移植物抗宿主病（cGVHD）诊断与治疗中国专家共识（2021年版）》基础上，参考国内外指南/共识和新近研究成果后制定，旨在为规范cGVHD的临床诊治提供指引。

一、cGVHD的发生机制和诊断评估

（一）cGVHD的发生机制

cGVHD主要病理生理过程为免疫炎症反应，常见和特征性的病理改变是纤维化。基于基础和临床研究，将cGVHD的发生分为3个阶段：组织损伤引起的早期炎症（第1阶段），慢性炎症引起的胸腺损伤及B细胞和T细胞免疫失调（第2阶段），最终导致组织纤维化（第3阶段）。值得注意的是，cGVHD的三个阶段通常是连续性事件，但是处于第1阶段的患者也可同时存在第2和第3阶段的状态[3]–[7]。

（二）cGVHD的诊断和临床评估

1. cGVHD的诊断：cGVHD的诊断主要依靠临床征象，类似于自身免疫性疾病，可以累及全身的任何一个或多个器官，最常累及的是皮肤、毛发、指甲、口腔、肝脏、眼睛、胃肠道、生殖器、关节筋膜或骨关节等[8]–[10]（见表1）。cGVHD的临床征象分为诊断性和区分性两种。诊断性征象包括：皮肤异色病、皮肤扁平苔藓、口腔或阴道苔藓、筋膜炎或关节痉挛等；区分性征象指只见于cGVHD而不见于急性移植物抗宿主病（aGVHD）的临床表现，包括皮肤色素脱失、指甲萎缩、脱发、口腔干燥、黏液腺囊肿、口腔溃疡、眼结膜干燥和多发性肌炎等。allo-HSCT后患者出现至少1项cGVHD的诊断性征象，或至少1项cGVHD的区分性征象，且伴有同一或其他器官支持cGVHD的辅助检查阳性（组织病理、实验室检查及肺功能实验等），均可诊断为cGVHD[11]–[12]。

**表1:** 慢性移植物抗宿主病（cGVHD）的临床征象（参考NIH 2014）[15]–[16]

受累器官或者部位	诊断性征象（诊断充分）	区分性征象（诊断不充分）	共同征象（急、慢性GVHD均可见）	非典型征象
皮肤	皮肤异色病、扁平苔藓样变、硬皮病	色素脱失	红斑、斑丘疹	汗腺功能障碍、鱼鳞病、毛发角化病、皮肤色素沉着减退、皮肤色素沉着过度
指甲		病甲、甲软化、甲脱离		
头发和体毛		脱发、斑秃		头发稀疏、斑秃、毛发粗糙或无光泽（无法用内分泌或其他因素解释）、早发白发
口腔	扁平苔藓样变、口腔活动受限	口干、黏液囊肿、溃疡、假膜^a^	牙龈炎、黏膜炎、红斑	
眼		角膜结膜炎^a^、Sicca综合征（泪腺功能障碍）		畏光、眶周色素沉着、眼睑炎（眼睑水肿、红斑）
生殖系统	扁平苔藓样、阴道/尿道挛缩	糜烂、龟裂、溃疡^a^		
消化道	食管网格形成、狭窄或硬化		厌食、恶心、腹泻	
肝脏			混合性肝炎	
肺	活检证实的支气管闭塞	经肺功能或影像学诊断的支气管闭塞		隐源性机化性肺炎、限制性肺疾病
肌肉，筋膜	筋膜炎、关节挛缩	肌炎和多发性肌炎		水肿、肌肉痉挛、关节炎或关节痛
造血系统			血小板减少、嗜酸性粒细胞增多、低或高丙种球蛋白血症、自身抗体形成	血小板减少、嗜酸性粒细胞增多、淋巴细胞减少、低或高丙种球蛋白血症、自身抗体形成、雷诺现象
其他			心包积液、胸腔积液、腹水	心包或胸腔积液、腹水、周围神经病变、肾病综合征、重症肌无力、心脏传导异常、心肌病

**注** ^a^所有情况必须排除感染、药物、肿瘤等因素

诊断需注意以下几点：①cGVHD以临床表现为主要诊断依据，但需排除感染、药物不良反应或第二肿瘤等其他疾病，建议必要时行组织活检明确诊断或进行专科会诊或多学科（MDT）综合诊治；②cGVHD早期征象不典型，应定期随访和密切观察。一旦出现晨僵、皮肤感觉异常、肌肉酸痛、不明原因低热、乏力或活动后喘息、不明原因消瘦、眼涩、口干、味觉异常、肝肾功能异常、感觉或运动轻度障碍、粪便性状改变及生殖系统异常变化等征象均需高度重视，积极开展诊断筛查[13]；③诊断指标并不等同于评价cGVHD严重性和治疗反应的指标[14]。

2. cGVHD的临床评估：cGVHD诊断明确后需要进行临床评估，以便对患者治疗指征和生存质量、预后进行判定，也是疗效评估和病情随访的重要依据。

（1）cGVHD严重程度分级：推荐根据八大受累器官（皮肤、口腔、眼、胃肠道、肝脏、肺、关节和筋膜、生殖器）的严重程度进行划分：0分指无症状；1分指没有严重的功能受损，对日常活动没有影响；2分指对日常活动有明显影响但无残疾；3分指对日常活动有严重影响伴有严重残疾。综合各项积分将cGVHD分为轻、中、重三类，反映严重程度[12]。轻度：1～2个器官最高1分（肺除外）；中度：3个及以上器官1分，或至少一个器官2分（肺除外），或肺1分。重度：至少一个器官3分，或肺2分或3分（表2）[15],[17]–[18]。

**表2:** 慢性移植物抗宿主病（cGVHD）的分级评分系统（参考NIH 2014）[11]–[12]

	0分	1分	2分	3分
功能评分：□KPS □ECOG □LPS	□无症状，活动完全不受限（ECOG 0分；KPS或LPS 100%）	□有症状，体力活动轻度受限（ECOG 1分；KPS或LPS 80%~90%）	□有症状，可自理，<50%时间卧床（ECOG 2分；KPS或LPS 60%~70%）	□有症状，生活自理受限，>50%时间卧床（ECOG 3~4分；KPS或LPS <60%）
皮肤、毛发、指甲 □斑丘疹，扁平苔藓样变 □丘疹，鳞屑样病变或鳞癣 □色素沉着□毛周角化 □红斑□红皮病 □皮肤异色病□硬化改变 □瘙痒症□毛发受累 □指甲受累	□无体表受累 □皮肤无硬化病变	□<18%体表面积	□19%~50%体表面积 □皮肤浅层硬化，未绷紧，可捏动	□>50%体表面积 □皮肤深层硬化 □皮肤绷紧，不可捏 □皮肤活动受限 □皮肤溃疡
口腔 □有□无 扁平苔藓样变	□无症状	□轻度症状，摄入不受限	□中度症状，摄入轻度受限	□严重症状，摄入明显受限
眼 □有□无 干燥性结膜炎	□无症状	□轻度干眼症（每日需要滴眼液<3次或无症状性干燥性角结膜炎）	□中度干眼症（每日需要滴眼液≥3次），不伴有视力受损	□严重干眼症，无法工作，视力丧失
胃肠道 □食管狭窄□吞咽困难 □恶心□呕吐□腹痛腹泻□体重下降	□无症状	□有症状，三个月内体重减轻<5%	□中到重度症状，体重减轻5%~15%，或中度腹泻，不妨碍日常生活	□体重减轻>15%，需要营养支持或食管扩张
肝脏	□总胆红素正常，ALT或碱性磷酸酶低于3倍正常值上限	□总胆红素正常，ALT在正常值上限3~5倍之间，或碱性磷酸酶高于3倍正常值上限	□总胆红素升高，但<51.3 µmol/L（3 mg/dl），或ALT高于5倍正常值上限	□总胆红素>51.3 µmol/L（3 mg/dl）
肺	□无症状 FEV1≥80%	□轻度症状（爬1楼气短） FEV1 60%~79%	□中度症状（平地活动气短）FEV1 40%~59%	□重度症状（静息气短，需吸氧）FEV1≤39%
关节和筋膜	□无症状	□肢体轻微僵直，不影响日常生活	□四肢至少1个关节僵硬，关节挛缩重度受限	□挛缩伴严重活动受限（不能系鞋带、系纽扣、穿衣等）
生殖系统	□无症状	□轻度症状，查体时无明显不适	□中度症状，检查时轻度不适	□严重症状
总体GVHD严重程度	□无GVHD	□轻度 1个或2个器官受累，得分不超过 1分，肺0分	□中度 3个及以上器官受累，得分不超过1分；或者至少有1个器官（不包括肺）得分2分；或肺1分	□重度 至少有1个器官得分3分；或肺 2分或3分

**注** FEV1：第1秒用力呼气容积；ECOG：美国东部肿瘤协作组评分；KPS：Karnofsky功能状态评分；LPS：Lansky功能状态评分

（2）cGVHD的预后危险分级：本共识推荐纳入12项危险因素用于cGVHD的预后评估（表3）。根据评分总数将危险度分为4组（Risk Group, RG）：RG1（0～3分），RG2（4～6分），RG3（7～9分），RG4（≥10分），评分越高，预后越差[19]–[20]。建议结合患者临床数据和靶器官受累的机器学习（ML），计算分析协助cGVHD生存风险预测评估。我国学者通过ML分析发现第1秒用力呼气容积（FEV1）<40％、肺炎、肺外cGVHD和呼吸衰竭是影响移植后闭塞性细支气管炎综合征（BOS）3年死亡率的独立危险因素，以上每个危险因素的存在赋1分（总分0～4分），基于此构建的FACT风险评分系统可识别高风险BOS患者[21]。

**表3:** 慢性移植物抗宿主病（cGVHD）预后危险评分系统（参考EBMT-NIH-CIBMTR工作组2018）

参数	危险度评分（分）
患者移植时年龄	
<30岁	0
30~59岁	1
≥60岁	2
早期aGVHD	
无	0
有	1
cGVHD与移植间隔时间	
≥5个月	0
<5个月	1
cGVHD发生时血清胆红素	
<34.2 µmol/L（2 mg/dl）	0
≥34.2 µmol/L（2 mg/dl）	2
cGVHD发生时的KPS(Karnofsky)功能状态评分	
≥80分	0
<80分	1
cGVHD发生时外周血小板计数	
≥100×10^9^/L	0
<100×10^9^/L	1
供者来源	
同胞间全相合/无关供者全相合或部分相合（1个位点不合）	0
其他相关/错配的无关供者（2个及以上位点不合）	1
移植时疾病状况^a^	
早期	0
中期	1
晚期	2
性别错配（供者/受者）	
男/男，男/女，女/女	0
女/男	1
GVHD预防	
环孢素A+甲氨蝶呤+其他	0
他克莫司+甲氨蝶呤+其他T细胞清除	1
cGVHD发生时外周血淋巴细胞计数	
≥1.0×10^9^/L	0
<1.0×10^9^/L	1
cGVHD发生时外周血嗜酸性粒细胞计数	
≥0.5×10^9^/L	0
<0.5×10^9^/L	1

**注** ^a^疾病状况的早期：AL-CR_1_、CML-CP、MDS-RA、MDS-RAS；中期：AL-CR_2_、CML-AP；晚期：白血病复发或诱导失败、CML-BP、MDS伴原始细胞增多。aGVHD：急性移植物抗宿主病

（3）cGVHD生物标志物：cGVHD的生物标志物包括细胞因子、趋化因子、microRNA、自身抗体、代谢产物等[22]，根据应用场景可分为：诊断、预测、预后、易感性/风险、治疗反应性生物标志物[23]（表4）。推荐将ST2、Reg3α、CXCL9、DDK3、MMP3作为辅助诊断的生物标志物[24]；靶器官特异性中，IL-8、IFN-γ、CXCL9及CCL17在眼部cGVHD患者泪液中表达增高[25]。需要重视的是，生物标志物的检测容易受其他因素影响，cGVHD生物标志物有待进一步探索，鼓励患者入组相关的临床研究。

**表4:** 慢性移植物抗宿主病（cGVHD）生物标志物类型（参考FDA-NlH生物标志物工作组2018）

类型	定义	应用	生物标志物
诊断生物标志物	用于探测或辅助诊断cGVHD或确定患者cGVHD亚型的生物标志物	辅助cGVHD诊断，为启动治疗时机提供参考	CXCL9、CXCL10、CCL17、CCL19、Reg3α、ST2、sBAFF，TNF-α、IFN-γ、TGF-β、IL-8、IL-10、IL-15
预测生物标志物	用于确定患者是否会从某项治疗中受益的生物标志物	决定何种cGVHD治疗对于患者是最理想的；作为临床危险因素使用以确定患者预防方案	–
预后生物标志物	用于预测中/重度cGVHD患者病情进展可能性的生物标志物	cGVHD发病时以及特殊cGVHD亚型中预测疾病进展可能性	MMP3、DKK3、ST2、CD163
易感性/风险生物标志物	用于确定目前无中/重度cGVHD临床征象的患者发生中/重的cGVHD的可能性的生物标志物	为启动cGVHD抢先治疗提供参考	Reg3α、L-纤维素、透明质酸、ST2、CXCL9、CXCL10、DDK3、MMP3
治疗反应生物标志物	用于表明接受治疗的患者已发生的潜在有益或有害的生物反应的生物标志物	确定治疗反应	sBAFF、IL-10、anti-PDGFR

二、cGVHD的预防

GVHD的发生与供受者性别/年龄、HLA相合程度、预处理方案、造血干细胞来源等因素相关。在造血干细胞移植中，cGVHD的预防一般是在aGVHD预防的基础上，对于GVHD的预防作为整体进行统筹，并不专门针对cGVHD而设。

（一）移植物来源选择

移植物来源是影响GVHD发生的重要因素。近年来，外周血干细胞（PBSC）成为主要移植物来源，PBSC中含有较多成熟的供者T细胞，在减少复发风险的同时也增加了GVHD发生率[26]。单倍体供者、无关供者相较同胞全相合供者更易引发GVHD[27]；而脐血造血干细胞移植患者的GVHD较轻[28]–[29]。在选择移植物来源时，建议充分评估患者的疾病状态、HLA相合等情况来选择合适的供者。

（二）免疫抑制药物

免疫抑制剂作用于T细胞增殖分化与激活的各个阶段，限制T细胞功能，抑制免疫反应。临床常用免疫抑制剂组合包括：

1. 钙调磷酸酶抑制剂（CNI）+甲氨蝶呤（MTX）+霉酚酸酯（MMF）/西罗莫司（SRL）：这一组合是allo-HSCT常用免疫抑制方案。CNI推荐环孢素A（CsA）或他克莫司（FK506）应用3～6个月，CsA有效谷浓度维持150～250 µg/L，FK506有效血浓度为7～12 µg/L，在CNI应用过程中严格监测肝肾功能，避免肝肾不良反应发生[30]–[31]；MTX：+1 d 15 mg/m^2^，+3 d 10 mg/m^2^、+6 d 10 mg/m^2^；HLA全相合无关供者移植或单倍体移植，建议在+11 d增加一剂MTX 15 mg/m^2^［32]；MMF 0.5 g每日2次，应用1～3个月。国内有研究显示，小剂量MMF（0.25 g每日2次，−1 d～+100 d）联合CsA、MTX对于HLA全相合无关供者移植后GVHD具有较好的预防效果[33]。有条件的单位建议进行MMF血药浓度监测（完全AUC/简化AUC目标浓度30～60 mg·h·L^−1^），根据浓度调整MMF用量[34]。西罗莫司作为MTX/MMF的替代用药，建议从移植前3 d开始用药，持续用药3～6个月，维持血药浓度5～15 µg/L。应用西罗莫司后，cGVHD发生率有不同水平下降，但重度cGVHD发生率与传统预防方案无差别[35]–[36]。

2. 抗胸腺细胞球蛋白（ATG）：ATG可去除移植物中T细胞，是预防和控制GVHD最直接的手段。体外去T虽然能预防GVHD，但降低移植物抗白血病效应（GVL），增加复发风险[37]，国内基本不推荐。我国单倍体移植、HLA全相合无关供者移植及部分HLA全相合同胞供者移植中，推荐ATG进行T细胞体内去除[38]，兔抗人胸腺细胞免疫球蛋白用量为1.5～2.5 mg·kg^−1^·d^−1^，−5 d～−2 d使用[39]；若选用兔抗人T细胞免疫球蛋白，用量一般增加1倍；也有移植中心使用猪抗人T细胞免疫球蛋白（25～30 mg·kg^−1^·d^−1^，−5 d～−2 d给药）。“北京方案”推荐ATG+CsA/FK506+MTX+MMF组合，对于供受者年龄>40岁的同胞全相合移植给予ATG 4.5 mg/kg（−3 d～−1 d应用），有助于降低cGVHD发生率[40]；“北京方案”推荐在单倍体移植中，兔抗人胸腺细胞免疫球蛋白降到7.5 mg/kg仍可有效预防cGVHD，巨细胞病毒（CMV）/EB病毒（EBV）相关死亡率下降[41]。此外，建议根据临床中疾病状态、移植类型不同和GVHD评估风险进行适当增减ATG用量。基于ATG预防改良方案的几项临床研究，有以下几种建议：小剂量ATG（6 mg/kg）联合低剂量移植后环磷酰胺（PTCy）（20 mg/kg，+3 d、+4 d）、MMF和CsA，cGVHD的2年累积发生率下降12.0％，该方案的循证依据最高[42]；在氟达拉滨/白消安/阿糖胞苷（FBA）基础上联用小剂量ATG（2.5 mg/kg，+8 d）和减低剂量PTCy（40 mg/kg，+3 d、+4 d）预防cGVHD，2年生存率提高21.3％[43]；ATG（5～9 mg/kg）基础上联合巴利昔单抗（Basiliximab）（20 mg，0 d、+4 d），3年cGVHD发生率为12.3％[44]。我国学者通过“北京方案”单倍体外周血造血干细胞移植体系的ATG体内药代动力学的监测获得了国人最佳ATG暴露范围，即活性ATG的总浓度-时间曲线下面积（AUC）为100～148.5 UE·d/ml，此区间内的患者植入稳定、GVHD发生率较低、CMV/EBV激活率亦较低。根据这一AUC范围对单倍体移植患者前瞻性干预ATG给药剂量，降低了移植后CMV和EBV再激活率，改善了移植后1年无病生存（DFS）率和1年总生存（OS）率[45]–[46]。

3. PTCy：对于HLA全相合移植患者，单独应用PTCy预防方案［+3 d、+4 d，静脉输注高剂量Cy（<50 mg/kg）］而不使用MTX/CNI常规预防方案，cGVHD发生率仅为7.0％[47]。对于外周血造血干细胞移植患者，PTCy预防cGVHD的发生率控制在20％以下[47]。对于HLA不全相合造血干细胞移植，建议在PTCy的基础上加用CsA、FK506、西罗莫司和ATG等药物，以提高GVHD的预防效果。有研究报道PTCy联合短疗程CsA方案（移植前3～5 d开始CsA 1.5 mg/kg每日2次静脉滴注，CsA血药浓度250～350 µg/L；无GVHD患者在第70天时停止使用CsA而非逐渐减量），2年cGVHD发生率为16.0％[48]。最新Ⅲ期临床研究显示，在全相合、7/8相合外周血干细胞移植中，PTCy联合FK506及MMF相比于标准方案MTX/FK506，1年无GVHD无复发生存（GRFS）率提升17.8％，cGVHD累积发生率下降13.2％[49]。PTCy的主要不良反应为骨髓抑制，还可引起膀胱刺激症、血尿、蛋白尿、肝功能损害和胃肠反应等[47],[50]。

（三）间充质干细胞（MSC）

MSC可通过促进调节性T细胞（Treg细胞）增殖活化，调控Th1/Th2比例发挥免疫调节作用[51]；也可通过上调CD27^+^记忆B细胞数量，降低血清B细胞激活因子（BAFF）水平和促进B细胞表面BAFF受体表达，诱导免疫耐受[52]。经MSC治疗的aGVHD患者后期cGVHD发生率降低[53]，近期我国已颁布《间充质干细胞防治移植物抗宿主病临床试验技术指导原则》。我国学者对MSC输注的剂量、时间和疗程的临床研究结果表明，移植后100 d给予MSC连续输注可预防单倍体造血干细胞移植后cGVHD，同时不增加白血病复发（推荐剂量1×10^6^/kg每月1次，共4次）[51]；MSC输注时间提前至移植后45 d，缩短输注间隔时间，可降低重度cGVHD发生率且提高GRFS率（推荐剂量1×10^6^/kg，每2周1次，共4次）[54]。移植后100 d MSC输注对cGVHD有明确预防疗效（肺部cGVHD保护效应更显著），移植后45 d MSC输注体现在生存获益（尤其是GRFS），可根据临床需求分层推荐。

三、cGVHD的治疗原则与一线治疗

（一）cGVHD的治疗原则和诊治流程

不是所有cGVHD的患者一旦确诊都需要全身治疗。建议根据NIH cGVHD的临床评估结果，轻度患者可观察或进行局部治疗；3个及以上器官受累或单个器官受累2分以上的中、重度患者需进行全身治疗[11],[55]。cGVHD的诊治流程见图1。

**图1:**
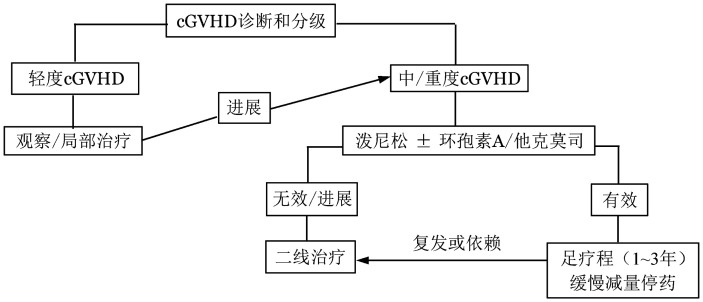
慢性移植物抗宿主病（cGVHD）的诊治流程 **注** 二线治疗具体药物根据病情个体化选择

（二）cGVHD的一线治疗

推荐糖皮质激素联合或不联合CNI作为cGVHD一线治疗的标准方案。以泼尼松为例，一般为1 mg·kg^−1^·d^−1^，单次服用；CsA 3～5 mg·kg^−1^·d^−1^，分2次口服，血药浓度维持150～250 µg/L；他克莫司0.1～0.3 mg·kg^−1^·d^−1^分2次口服或0.01～0.05 mg/kg持续静滴，血药浓度维持7～12 µg/L。一线治疗的有效率约为50％。

如果一线治疗有效，cGVHD症状得到有效控制后，糖皮质激素应逐渐减量。目前尚无公认的糖皮质激素减量方案，推荐把握以下原则：缓慢减量、足够疗程，尽量使用足以控制GVHD症状的剂量。建议采用每2周梯度递减约原剂量的20％～30％，具体遵照隔日减量法，如先减偶数天的服药剂量[56]；激素联合CsA等CNI治疗中，建议先减激素，其他免疫抑制剂每2～4周减量1次，3～9个月时间减停1种，免疫抑制剂治疗的中位时间应该足够长，建议1～3年。

具有糖皮质激素禁忌症或者口服不耐受的患者建议选择无糖皮质激素方案，包括CNI（71.4％）、芦可替尼（61.2％）和体外光分离置换疗法（ECP）（57.1％），但仅不到20％的患者适用[57]。对于“糖皮质激素+X”的一线治疗模式建议开展相关的临床研究：奥法木单抗（Ofatumumab）联合糖皮质激素治疗cGVHD的6个月总反应率（ORR）为62.5％，泼尼松剂量由1 mg·kg^−1^·d^−1^减至0.14 mg·kg^−1^·d^−1^，提高了24个月停用糖皮质激素的成功率[58]；芦可替尼联合糖皮质激素在儿童cGVHD初始治疗中起效快，耐受性良好，对以关节、肝脏、肺为靶器官的cGVHD疗效显著[59]；利妥昔单抗联合糖皮质激素治疗cGVHD的12个月ORR为83.0％，3个月后83％的患者糖皮质激素剂量减少30％以上，74％的患者12个月后停用糖皮质激素[60]；小剂量MTX（5 mg/m^2^）联合糖皮质激素组移植后1年中、重度cGHVD的发生率降为11.0％[61]；伊布替尼（420 mg每日1次）联合糖皮质激素与单用糖皮质激素一线治疗的48周反应率差异无统计学意义[62]。需进一步临床研究增加循证医学证据。

四、cGVHD的二线治疗

（一）启动二线治疗的时机

临床出现以下情况推荐启动二线治疗：①既往累及的器官损伤加重；②出现新的器官受累；③正规用药1个月症状体征没有改善（如果单用糖皮质激素治疗，初始治疗2周有进展，6～8周无改善，考虑糖皮质激素耐药）；④2个月时，泼尼松减量不能低于1.0 mg·kg^−1^·d^−1^［17]。与aGVHD不同，建议更换二线治疗药物后需要给予足够的观察期，不要急于短时间换药，一般观察8～12周，除非4周内病情明显进展时才能考虑再次更换其他二线药物。

（二）常用的二线治疗方案

二线治疗目前尚无标准的优选治疗方案，各种二线治疗药物可以互换，推荐可依据个体化状况和靶器官特点尝试选择以下药物和措施[17]。

1. 小剂量MTX：用于allo-HSCT后GVHD的预防和aGVHD治疗，具有安全、有效的优点。推荐小剂量MTX方案用于cGVHD的一线治疗和难治性cGVHD的挽救治疗，剂量为5～10 mg/m^2^，第1、3（或4）、8天给药，此后每周1次，直至cGVHD症状缓解或不能耐受不良反应；如果白细胞计数<2×10^9^/L或血小板计数<50×10^9^/L，可减至5 mg/m^2^。MTX治疗cGVHD的总缓解率为76.2％～83.0％，局限型和皮肤型cGVHD缓解率最高，不良反应可接受[63]–[64]。欧洲cGVHD诊断治疗指南已将此MTX方案列为糖皮质激素耐药或不耐受cGVHD患者的重要挽救治疗方案（5～10 mg/m^2^每周1次，静脉给药）[65]。

2. 芦可替尼：推荐用于治疗糖皮质激素或其他系统治疗应答不充分的12岁及以上cGVHD患者[10],[66]。推荐剂量10 mg每日2次[67]，也可2.5～5 mg每日2次[68]。对于多重耐药cGVHD，芦可替尼5～10 mg每日2次治疗的总有效率为70.7％～78.0％[69]–[71]。长时间使用需注意感染的发生，特别是病毒（如CMV）再激活的问题，此外贫血、血小板减少也是芦可替尼较常见的不良反应，发生率为12.7％～15.2％[67]，在使用过程中需要注意观察。合用三唑类抗真菌药物可增加芦可替尼血药浓度和不良反应[72]。

3.甲磺酸贝舒地尔（Belumosudil）：甲磺酸贝舒地尔为ROCK2抑制剂，推荐用于治疗糖皮质激素或其他系统治疗应答不充分的12岁以上cGVHD患者，剂量为200 mg每日1次，ORR为65.0％～74.0％。当与强效CYP3A诱导剂或质子泵抑制剂联合用药时，剂量可增加至200 mg每日2次，ORR为69.0％～77.0％，治疗关节筋膜、上消化道、肝脏的ORR分别为77.8％、66.7％和66.7％；治疗BOS的ORR 32.0％，其中完全缓解（CR）率15.0％[73]–[75]。

4. 西罗莫司：最先用于实体器官移植后排斥预防及自身免疫性疾病[76]，后被用于难治性cGVHD的治疗[77]–[78]。建议西罗莫司2 mg/d口服，首剂加倍。体重低于40 kg则采用1 mg·m^−2^·d^−1^。有效治疗浓度5～15 µg/L，维持治疗时间3～6个月或cGVHD症状明显缓解后减量停药。西罗莫司与CNI联用具有协同并降低肾毒性的优点，1年ORR为59.3％，比较适合cGVHD长时间联合使用[79]。使用期间监测血药浓度和相关不良反应（口腔黏膜溃疡、高脂血症和骨髓毒性等）。除发挥免疫抑制作用外，西罗莫司尚具有抗纤维化，抗肿瘤及抗病毒活性作用（可用于预防CMV、EBV感染）[80]。

5. 伊马替尼：伊马替尼可调节T细胞、B细胞而起到免疫抑制作用[81]。建议伊马替尼应用于合并纤维化的cGVHD治疗。起始剂量100 mg/d，根据治疗效果和不良反应调整用量（最大量400 mg/d）[82]。治疗总缓解率为36.0％～70.0％，合并肝脏、肺部、皮肤病变患者显示出更好的治疗效果[83]–[84]。伊马替尼常见不良反应包括白细胞减少、血小板减少、水肿、皮疹，停药、对症处理后可改善[85]–[86]。

6. 利妥昔单抗：利妥昔单抗治疗难治性cGVHD的疗效在65.0％左右，对合并血小板减少症、硬皮病、皮肤病变、风湿性疾病的cGVHD患者效果更佳，建议用量375 mg/m^2^每周1次，连用4周[87]。利妥昔单抗联合MMF、FK506或西罗莫司的三联疗法，总缓解率达88.0％，2年生存率82.0％[88]，为替代糖皮质激素、减少不良反应、降低cGVHD治疗相关死亡率提供参考。

7. 伊布替尼：推荐应用于cGVHD的二线治疗，推荐剂量420 mg/d，总缓解率为67％。与糖皮质激素合用可显著减少糖皮质激素用量、延长中位缓解时间[89]。

8. MMF：MMF主要与CsA、MTX和（或）ATG联用以预防GVHD，也用于难治性cGVHD的挽救治疗，总缓解率为69.1％～72.0％，不良反应可接受[90]–[91]。目前尚无充分临床证据显示MMF在cGVHD的一线三联疗法（糖皮质激素+CNI+MMF）中发挥显著作用，糖皮质激素或CsA减量时可适当加用MMF，以减少cGVHD症状反复[92]。

9. MSC：MSC治疗难治性GVHD的效果还需要积累足够的循证医学证据，推荐剂量为1×10^6^/kg每2周1次，共2～4次，缓解率为57.1％～73.7％，对口腔黏膜、胃肠道、肝脏和皮肤病变治疗效果最佳[93]–[94]；在治疗眼部cGVHD方面MSC被认为有较好疗效，MSC外泌体（UC-MSC-exo）治疗难治性cGVHD干眼症，建议滴眼治疗（10 µg/50 µl，每眼每日4次，持续2周），能够有效缓解干眼症状[95]；我国学者采用羊膜上皮干细胞（hAESC）滴眼液治疗眼部cGVHD（1×10^6^细胞/ml，每眼每次2滴，每日4次，共6周），可有效缓解干眼症状、减轻角膜损伤[96]–[97]。

10. 沙利度胺及其结构类似物：在一项难治性成人cGVHD的Ⅱ期临床研究中，推荐治疗剂量为200 mg/d，治疗2、6、12个月的ORR分别为83％、88％、85％[98]。联合泼尼松/CsA并不能提高cGVHD治疗效果，且便秘、失眠、外周神经病变和血液学毒性等不良反应发生率较高，25.9％～92.0％的患者提前终止治疗[99]。因此，目前观点认为沙利度胺仅作为难治性cGVHD的备选和辅助治疗方案，并需要根据患者在治疗过程中的疗效和不良反应调整用量。泊马度胺作为沙利度胺结构类似物，建议剂量0.5 mg每日1次口服，6个月ORR达67％，对皮肤和关节受累显示出较好的治疗效果。常见不良反应包括淋巴细胞减少、感染和疲劳[100]。

11. 小剂量IL-2：建议小剂量IL-2应用于糖皮质激素抵抗型cGVHD（SR-cGVHD）的二线治疗（1×10^6^ IU·m^−2^·d^−1^，疗程8～12周），治疗4周后起效，有效率为52％～61％。常见不良反应包括发热、乏力和骨关节疼痛，均为Ⅰ～Ⅱ级[101]–[102]。低剂量IL-2（LD IL-2）治疗SR-cGVHD，8、12周后的总有效率分别为48.6％、53.3％，在所有受累器官中，肝脏特异性反应率最高（66.7％）[103]。

12. ECP：ECP是一种提取循环细胞后在体外进行长波紫外线照射的方法[104]，安全性高且不影响移植物抗白血病作用，推荐用于SR-cGVHD[105]。治疗缓解率达80％，成人与儿童缓解率无差异，适用于皮肤、口腔、肝脏病变者[106]。ECP治疗SR-cGVHD建议每周1次，反应率为87％，39％的患者达CR[107]。

13. 其他新药：①艾克利单抗（Axatilimab）：建议治疗剂量1 mg/kg每2周1次，ORR为82％，常见不良反应包括乏力、恶心、外周性水肿[108]；②阿巴西普（Abatacept），建议10 mg/kg，共6次，57.0％的患者肺获益最明显[109]；③福坦替尼（Fostamatinib）：建议100 mg每日2次，总缓解率为77.0％[110]；④巴利昔单抗（Basiliximab）：建议20 mg第1、3、8天各1次，之后每周重复1次，在肝脏cGVHD诊断后4周内接受治疗患者的ORR达83.3％[111]。上述几种药物的临床使用较少，尚需要进一步临床研究。

（三）建议根据cGVHD受累靶器官选择治疗方案

建议根据靶器官实际受累情况选择针对性治疗方法，根据不同受累器官对治疗的反应性，建议皮肤受累患者选择芦可替尼（ORR 59％）[59]、甲磺酸贝舒地尔（ORR 40％）[74]、伊马替尼（ORR 34.8％～46.0％）[112]–[113]、ECP（ORR 51％～91％）[107],[114]治疗；皮肤硬化型建议选择芦可替尼（ORR 77％）[115]、ECP（ORR 58％）[114]、艾克利单抗（ORR 84％）[108]治疗；肺受累推荐选择阿巴西普（ORR 57％）[109]、芦可替尼（ORR 33％～67％）[59],[115]、甲磺酸贝舒地尔（ORR 32.0％）[116]治疗；肝脏受累推荐选择芦可替尼（ORR 64％～80％）[70],[117]、ECP（ORR 80％～93％）[107],[118]、小剂量IL-2（ORR 66.7％）[103]、伊马替尼（ORR 41.0％～66.7％）[112]–[113]、阿巴西普（ORR 54％）[109]治疗。上述药物均是临床研究结果，cGVHD器官特异性治疗药物仍需进一步临床研究与验证[119]。

（四）康复治疗

康复治疗的目标是减轻症状、提高cGVHD患者生活质量。针对cGVHD导致的肌肉疼痛、关节僵硬等症状，建议采用物理治疗和康复训练帮助患者维持或改善日常生活活动。针对不同cGVHD靶器官的康复治疗，有利于改善cGVHD硬皮病症状和疤痕导致的关节活动受限；改善BOS患者的主观呼吸困难症状和运动耐受性[120]；康复治疗也能预防cGVHD治疗（如糖皮质激素、CNI）导致的骨密度降低、股骨头坏死和肌肉萎缩。需要强调，康复治疗需要个体化，治疗决策应该由专业的医疗团队根据患者的具体情况进行制定。患者和其家人也应积极参与医疗团队保持沟通，共同制定治疗计划[121]。

五、cGVHD患者的生活质量评估

cGVHD的发生和发展与HSCT后健康相关生活质量（HRQoL）密切相关，需要重视[122]–[123]。HRQoL和症状均为常用的患者报告结局（PRO）[124]，但缺乏标准的患者报告结局指标（PROM）评估和测量，也缺乏中国患者评价验证的推荐。现在认为李氏症状学量表（Lee symptom scale, LSS）是唯一经过验证的cGVHD特异性测量工具，SF-36、FACT-BMT为NIH工作组推荐的反应测量工具[18]；此外，HAP、EORTC QLQ-C30、EQ-5D-5L等其他量表也正在cGVHD患者队列中开展相关验证[125]。我国学者通过LSS评估cGVHD患者症状负荷、SF-36及EQ-5D-5L评估QoL，认为cGVHD患者的主要症状负荷来源为眼、心理和口腔，且以精神健康总评（MCS）受损为主；影响QoL重要的因素为总体症状负荷、眼NIH严重程度分级以及口腔症状负荷。因此，本共识建议将LSS作为症状负担评估工具[126]。

六、总结

allo-HSCT后cGVHD发生与多种因素相关：移植类型、供受者HLA相合程度、患者年龄及一般情况等。cGVHD可以累及全身的任何一个或多个器官，病理机制复杂，临床表现多样，以造血干细胞移植专科医生为主导的MDT诊治模式有利于cGVHD的诊断、受损器官评分、危险度分层和总体治疗方案制定。未来的发展方向应关注：①增加门诊随访，及时临床评估；②应用生物标志物进行综合评估并指导cGVHD精准选药；③新型药物或药物联合使用治疗SR-cGVHD；④cGVHD慢病全程化管理，器官动态评估；⑤制定中国cGVHD患者QoL量表监测生存质量；⑥cGVHD基于糖皮质激素的优化一线治疗方案，努力实现无糖皮质激素的一线治疗。cGVHD治疗是一个长期的医疗过程，需要加强医患沟通和患者教育，让医患双方都足够重视cGVHD规范治疗和随访管理。
